# Clinical experience with a same‐day simulation and treatment program for stereotactic radiation therapy on a C‐arm linac

**DOI:** 10.1002/acm2.70449

**Published:** 2026-01-02

**Authors:** Michalis Aristophanous, Sernger Shen, Dylan G. Hsu, Dongxu Wang, Ase Ballangrud, Jean M. Moran, Anyi Li, Sean M. McBride, Daniel Gomez, Luke R. G. Pike, Kathryn Beal, Jonathan T. Yang, Laura Cervino

**Affiliations:** ^1^ Department of Medical Physics Memorial Sloan Kettering Cancer Center New York New York USA; ^2^ Department of Radiation Oncology Memorial Sloan Kettering Cancer Center New York New York USA

**Keywords:** SRS, SBRT, same‐day

## Abstract

**Purpose:**

We report our experience with the implementation of a same‐day simulation and treatment C‐arm linear accelerator (linac)‐based stereotactic program for patients with intracranial and extracranial metastatic disease.

**Methods:**

Between May 2021 and October 2023, patients were treated in our same‐day program with linac‐based SRS/SBRT. Two slots per week were offered. Patients with expedited clinical needs, able to undergo SRS/SBRT simulation and treatment, were considered. Extracranial treatments were required to meet standards for automated intensity modulated radiation therapy (IMRT) optimization. Intracranial treatments were limited to 1–3 lesions and 1–2 isocenters. The day before treatment, the patient needed to be identified, and any diagnostic imaging had to be available for the physician and dosimetrist to discuss the plan. On the day of treatment, simulation was scheduled for 8 AM and treatment at 4 PM by default, with the goal to complete treatment by 6 PM. We analyzed information about each patient's treatment plan and time spent on each step of the workflow.

**Results:**

Ninety‐seven patients followed our same‐day workflow and were included in the analysis. Seventy‐five patients received intracranial SRS (57% to 1 lesion), while 22 patients received extracranial treatments (50% to the extremities). Simulation often required additional time to be completed, finishing a median 18 min (IQR 5–40) after the goal end time. The median time between simulation completion and end of the same‐day treatment was 7.8 h (IQR 7.4–8.6). Treatment technique and the number of target volumes had a significant impact on planning time. The median treatment end time was 5:13 PM (IQR 4:46 PM–6:01 PM), with 74% ending by 6 PM.

**Conclusions:**

A linac‐based program to treat patients with SRS/SBRT in an expedited fashion was established and successfully treated patients in a same‐day timeline. Careful selection of planning techniques to limit plan complexity and adding automation in time‐consuming parts of the process are crucial when developing expedited workflows.

## 1 INTRODUCTION

Stereotactic treatments for metastatic disease have seen a dramatic increase in the last 10 years.^1^, ^2^ In general, there has been shift in practice away from simple parallel‐opposed beams towards focal targeting with SRS for intracranial disease and stereotactic body radiation therapy (SBRT) for extracranial disease. These modalities deliver higher doses with tighter spatial margins to achieve improved local control with fewer toxicities.^3^, ^4^ At the same time, the ability to conduct the process from simulation to treatment in an expedited manner has diminished as a result of the increased complexity of treatment planning.

Early SRS necessitated the involvement of a neurosurgeon and was delivered with frames fixed to the patient's head.^5^ The paradigm was one lesion, one isocenter, and relied on simpler planning techniques to make it possible to complete in a single day. At the same time as frameless SRS was introduced, targeting multiple lesions in one session became more widespread, driving the adoption of more complex planning techniques. The Gamma Knife is one of the oldest technologies^6^ that necessitates a one‐day procedure; however, it requires specialized equipment and handling radioactive materials, posing logistical challenges for hospitals. On the other hand, for extracranial metastases, the treatment of choice was often parallel‐opposed beams with simple dosimetric calculations,^7^ which again, due to the simplicity of the plans, permitted simulation and treatment on the same day. SBRT for extracranial metastases is increasingly being utilized and has been shown to improve survival and has excellent local control.^8^, ^9^ Conventional C‐arm linacs do not currently come with the necessary infrastructure to support same‐day procedures for SRS and SBRT. The planning typically can take several hours, and the workflows are not straightforward, necessitating several steps to prepare the plan for treatment. MR and CBCT‐based adaptive radiotherapy machines are now enabling faster treatment starts with templated planning and simulation‐less workflows;^10^, ^11^ however, they also rely on dedicated machines and software that are not universally available.

Despite the increasing need for more complicated plans to treat metastatic cancer, the need to initiate treatment quickly for cancer patients has not diminished. In fact, Hanna et al. quantified the effect of treatment delays, and they found that a 4‐week delay is associated with increased mortality for seven primary cancers.^12^ In the context of metastatic disease, being able to treat quickly is also of utmost importance. Seeing tumors grow beyond their delineated borders between the time of simulation or diagnostic imaging and treatment is not uncommon.^13^, ^14^ In addition, patients may be in significant pain, necessitating expedited palliative intervention with radiation therapy. Often, these kinds of scenarios may result in sub‐optimal, yet simpler plans, rather than an expedited course of SBRT that can better control the disease.^15^ Chowdry et al. completed a quality initiative study to determine turnaround times from simulation to treatment, which showed that two weeks were needed to turn around plans of increased complexity for intensity‐modulated radiation therapy (IMRT) or volumetric modulated arc therapy (VMAT).^16^

With this program, we aimed to offer both SRS and SBRT treatments on a conventional C‐arm LINAC in an expedited manner—specifically, to complete the entire simulation‐to‐treatment workflow on the same day. We also aimed to offer this to as many patients as possible who were in need of SBRT for oligometastases or brain SRS, by balancing accessibility with feasibility, as often plan complexity may necessitate additional time to complete all the steps in the process in a safe manner. In this work, we present the same‐day SRS/SBRT program in detail, and the clinical experience after two and a half years of implementation. We show the population of patients treated within the program, analyze the timelines and delays, and finally discuss the significance of our findings.

## 2 METHODS

### 2.1 Patient selection

Between May 2021 and October 2023, patients needing expedited treatment, as determined by their primary physician, were added to the program if they satisfied the eligibility criteria summarized in Table 1.

**TABLE 1:** Summary of criteria for patients that can be added to the same‐day program.

Intracranial metastases	Extracranial metastases
Maximum of three lesions	Maximum of one site
Same fractionation for each lesion	No simultaneous integrated boost (dose‐painting)
No more than two isocenters are needed	Cases can be auto‐planned for IMRT

### 2.2 Treatment planning and delivery

The treatment planning team included four planners who were assigned on a rotating basis on the day with an available accelerated SRS/SBRT slot. Their planning load was adjusted to ensure they had no other treatment plans due on that day, so they would be able to devote much of the day to the same‐day case.

Intracranial SRS planning was completed in the Eclipse treatment planning system (Varian Medical Systems, Palo Alto, CA). Planning was performed with VMAT or dynamic conformal arc (DCA), as needed. For patients treated from May 2021 until June 2022, the CDR Freedom X board with the Intuition Mold and thermoplastic mask was used for simulation to immobilize the head (CDR Systems, Calgary, AB). In the summer of 2022, we transitioned to the Encompass immobilization system (CQ Medical, Avondale, PA) to enable Varian's Hyperarc module for optimization. Contouring of volumes was completed in MIM Maestro (MIM Software Inc., Cleveland, OH) with autosegmentation and organs‐at‐risk (OARs) using an in‐house solution^17^ and MD creation of target volumes. The planning CT and structure set were then exported to Eclipse. An automatic in‐house developed Application Programming Interface (API) script^18^ was used to place optimal isocenters based on lesion distance, and for manual VMAT planning, it also optimizes collimator angles, and generates plans with our standard naming convention with beams set up ready for optimization.^19^ For cases where Hyperarc was used, the planner used the lesion grouping from the script and the Hyperarc tools to generate the beam template and optimize the collimator angles. For optimization, the standard or Hyperarc tools were used, and plan quality was evaluated using an in‐house script to calculate the gradient index and the conformity index^20^, ^21^

For extracranial SBRT, patients were immobilized using the CDR Freedom X board. The majority of these plans were automatically optimized in around 20 min for fixed‐gantry IMRT using the Expedited Constrained Hierarchical Optimization (ECHO) system*.^22^ A templated plan with 9 posterior beams is imported into Eclipse, and once the aperture is fit to the target, ECHO is launched via an API script. The optimization is performed at an external server, and the fluences when ready are imported back into Eclipse for the final dose calculation.

Patients were monitored on‐treatment with surface‐guided imaging using AlignRT (Vision RT Ltd., London, UK) for intracranial SRS treatments,^23^ and ExacTrac (BrainLab USA, Moorestone, NJ),^24^ or Intra‐fraction Motion Review (IMR) (Varian Medical Systems) for extracranial treatments. Patients were set up for each fraction using on‐board imaging with kV radiographs and cone‐beam CT (CBCT).

### 2.3 Workflow and timelines

#### 2.3.1 Day before treatment

**FIGURE 1:**
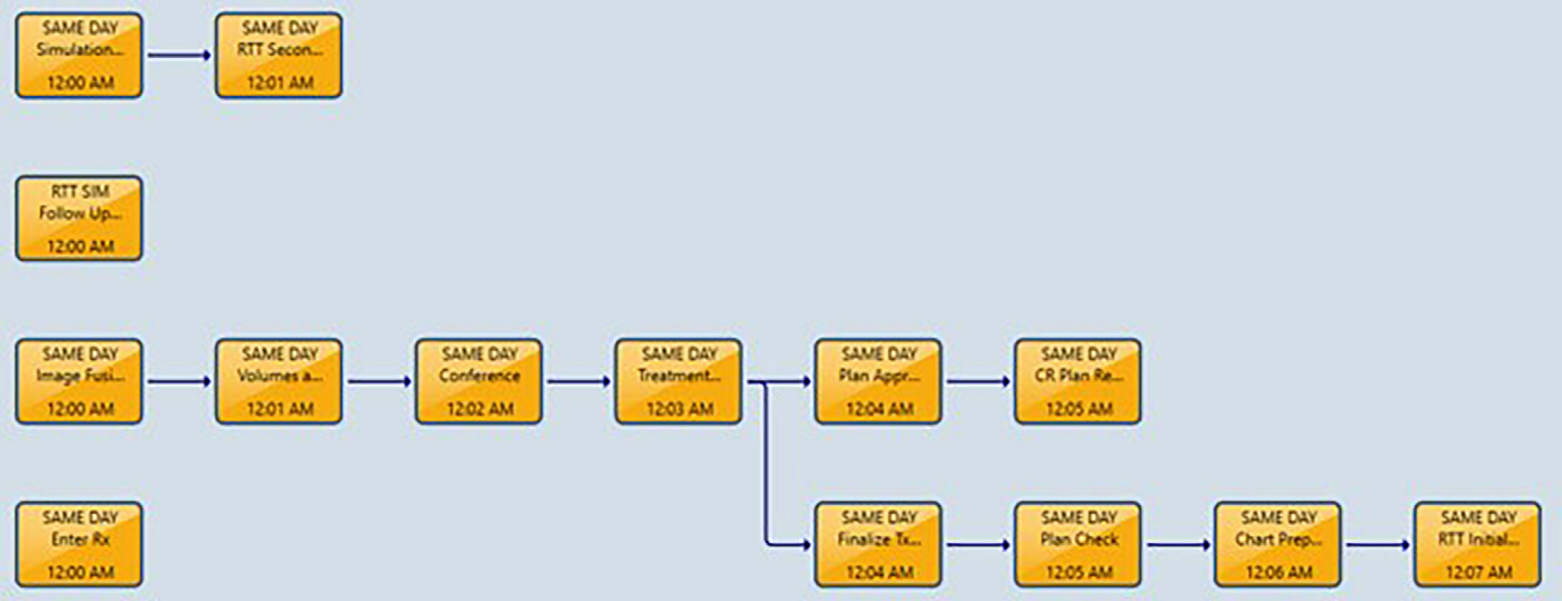
The workflow is shown through the ARIA care path.

To optimize efficiency, ensure quality, and avoid delays, certain tasks must be completed the day before treatment. First, once the patient is identified, the name and medical record number are entered by the clinical team in a calendar to reserve the spot. The physics team is notified through this calendar and completes a pre‐check to ensure the patient meets the criteria for the same‐day program and that all necessary imaging for fusions is available. The planner then contacts the physician to complete a conference, to discuss the case, and receive directions on planning priorities. The planner then drops a CarePath in ARIA (Varian Medical Systems), which is used in our clinic to track tasks related to a patient's treatment planning process. Figure 1 shows the workflow on the simulation and treatment day as it is built into this care path.

#### 2.3.2 Same‐day treatment day timeline

On the day of treatment, the patient has an appointment with the nursing team at 7:30 AM to check that they are ready for treatment. The simulation is scheduled for 1 h beginning at 8 AM; this typically involves making custom immobilization, setting triangulation markers, and conducting a CT scan with IV contrast. The planner then has a half‐hour to complete the image fusions (typically with MRI) with input from the physician as needed. By 10 AM, the physician finishes treatment volumes and reviews autosegmented OAR contours or creates missing OARs, and at 10 AM, the physician and planner have a quick conference call to discuss any updates to the guidance based on the imaging and contours. Approximately 3.5 h are allotted for planning, with the goal of physician plan approval by 2 PM. The plan is then finalized by making sure that all the documentation is printed, safety‐critical tasks like patient‐specific QA are completed or scheduled, motion monitoring is configured as needed, and any important information about the plan or patient setup is communicated to the therapists on the treatment machine. The plan is then sent to the physicist for plan check, which is intended to be completed by 3:30 PM. Our in‐house developed plan checking tool, which automates many of the checks required during physics plan check, greatly improves speed and efficiency in our plan checking process.^25^ Once the plan check is complete, any necessary patient‐specific QA needs to be run by 4 PM. At our institution, patient‐specific QA is performed for single fraction treatments using field‐by‐field EPID dosimetry using gamma analysis at 3%/2 mm evaluated at the 95% passing level. For hypofractionated treatments, no pre‐treatment patient‐specific QA is performed. A trajectory log file analysis is run every evening for both single and hypo‐fractionated treatments.^26^, ^27^ Treatment itself depends on machine scheduling and availability, but is typically scheduled between 4 PM–6 PM. Patients who were treated as inpatients were transported back to their rooms until the time of their treatment appointment, and patients who were treated as outpatients were given their treatment appointment time and asked to return in time for their treatment. A detailed description of the timeline is shown in Table 2.

**TABLE 2:** The same day timeline.

Day ‐1		Day 0	
Time	Task	Time	Task
1 PM	Identify patient	7:30–8:00 AM	Patient meets with nurses prior to CT sim
1 PM	Complete MRI (if applicable)	8:00–9:00 AM	CT sim w/ IV contrast, if indicated
5 PM	MD/planner conference	9:00–9:30 AM	Fusion (if applicable)
		9:00–10:00 AM	MD completes volumes in MIM
		10:00–10:15 AM	MD/planner conference
		10:15–1:45 PM	Treatment planning
		1:45–2:00 PM	Plan review by MD
		2:00–2:30 PM	Plan Finalization
		2:30–3:30 PM	Plan checking
		3:30–4:00 PM	Delivery and review of patient specific QA
		4:00–6:00 PM	Treatment

**FIGURE 2:**
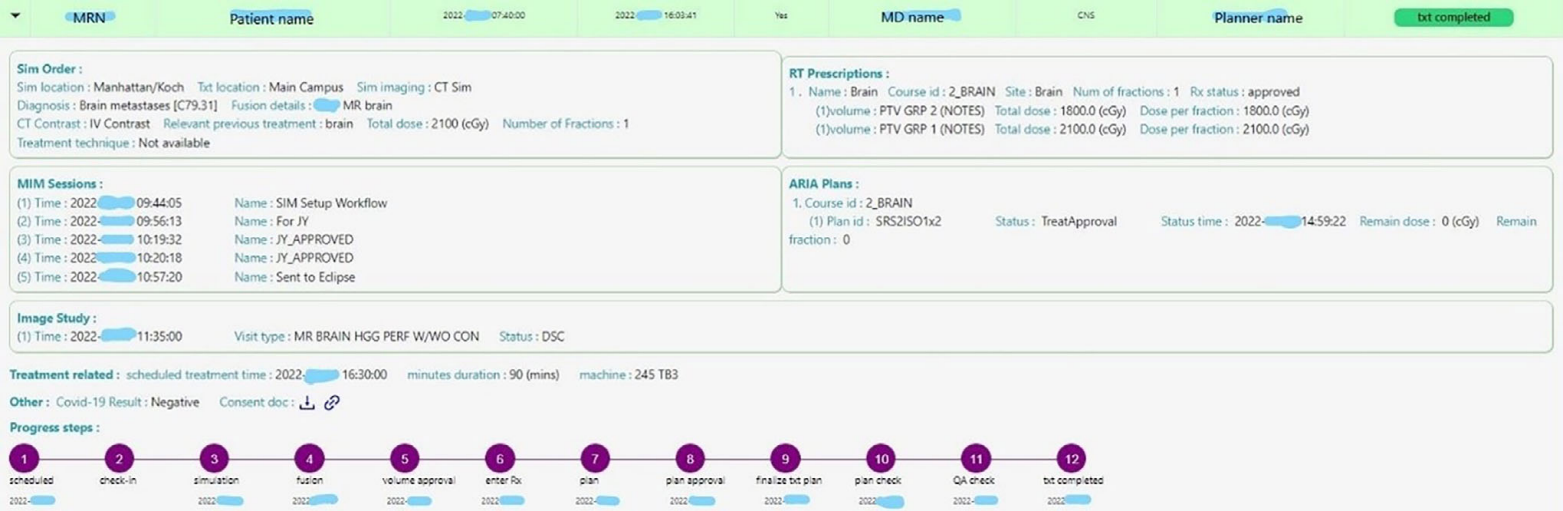
The same‐day dashboard to show the progress of the process.

To assist our same‐day team to stay within the established timelines, we built a dashboard that collects information from various systems (electronic medical record, ARIA/Eclipse, MIM Maestro) and displays the collected information in real time. The physics team of the day accessed the dashboard regularly to get updates on the status throughout the day. An example of the dashboard is shown in Figure 2. At the same time, in the morning of the procedure, a chat is initiated between all involved members of the team for real‐time updates. A formal escalation protocol that involves pages and emails is activated if responses are delayed.

### 2.4 Analysis

We collected demographic and treatment information for patients treated with this same‐day workflow. In addition, we collected completion time point information from the CarePath and dashboard, which was based on task completion time in the ARIA information system, and calculated the task duration based on the task completion times. Specifically, we looked at: simulation completion time; the duration of fusion, volume, planning, plan review and approval, plan finalization, plan check and QA; and treatment completion time. Differences in completion times and time durations were analyzed using the Student's *t*‐test. Correlations between durations and task completion times were quantified using the Pearson correlation coefficient. Comparisons were evaluated at the 0.05 significance level.

## 3 RESULTS

Of the 145 patients that were added to the same‐day program, 97 were included and analyzed. We excluded 48 patients from the analysis because they did not follow the same‐day timeline as it was laid out. In most of these excluded cases (36), the patients were simulated the day before. The distribution of treatment sites for extracranial treatment and lesion numbers for intracranial treatment are shown in Table 3. 75 patients received intracranial SRS, while 22 patients received extracranial SBRT. 61 patients (63%) received single‐fraction treatment, while 36 (37%) received hypofractionated radiotherapy.

**TABLE 3:** Patient characteristics.

	Number of lesions	Number of patients	Percent single fraction	Percent single isocenter
**Intracranial treatment**	1	43	77% (33)	100% (43)
	2	20	50% (10)	75% (15)
	3	7	43% (3)	86% (6)
	4	5	40% (2)	80% (4)
	**Subsite**			
**Extracranial treatment**	Extremity	11	55% (6)	
	Pelvis	8	75% (6)	
	Torso/HN	3	33% (1)	

Out of all the treatments, only seven (7%) were intracranial treatments requiring two isocenters. Among intracranial treatments, 65 (87%) were treated with VMAT, nine (12%) were treated with DCA, and one patient had two lesions treated with VMAT and DCA, respectively. Twenty extracranial SBRT treatments (91%) were auto‐planned for fixed‐gantry IMRT using ECHO; the two remaining were manually planned for VMAT.

**FIGURE 3:**
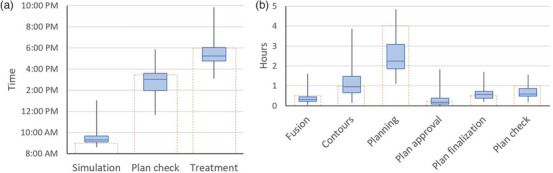
Box plot of (a) the simulation, plan check, and treatment end times, and (b) the distribution of duration time for different tasks in the planning process. The dotted bars on the two plots show the goal end times (a) and the goal task duration (b). A box plot shows the median by the horizontal line, the 1st and 3rd IQR with box bounds, and the full range with the whiskers.

Figure 3a shows the time that the simulation, plan check, and treatment delivery were completed. The median time for simulation completion was 9:18 AM (IQR 9:05–9:40), while the median time for completion of treatment delivery was 5:35 PM (IQR 4:46–6:01). The median time for plan checks completion was 3:01 PM (IQR 1:57–3:36). The simulation was completed by the 9 AM goal time for 12% of the cases and the treatment was completed by the 6 PM goal time for 74% of cases. 95% of patients finished treatment by 7 PM, and all but one patient finished by 8 PM. The earliest treatment completion time was 3:06 PM. The time it took for planning had a moderately significant correlation with the treatment end time (0.39, *p*‐value < 0.001), followed by the time to complete volumes (0.32, *p*‐value = 0.001), and time to prepare image fusions (*p*‐value = 0.01). The simulation end time did not have a significant correlation with treatment end time (0.16, *p*‐value = 0.1). Figure 3b shows the time it took to complete some of the intermediate steps in the process. The goal to complete the fusion, planning, and plan check within 1/2 h, 4 h, and 1 h, respectively, was met at least 80% of the time for all patients. On the other hand, the goal to complete contours, plan approval, and plan finalization within 1 h, 15 min, and 1/2 h, respectively, was only met 40%–60% of the time. Finally, the 90th percentile duration for each task was: 40 min for image fusions, 2.4 h for volume delineation, 4.1 h for treatment planning, 35 min for physician plan approval, 57 min for plan finalization, and 1.1 h for physics plan check.

**FIGURE 4:**
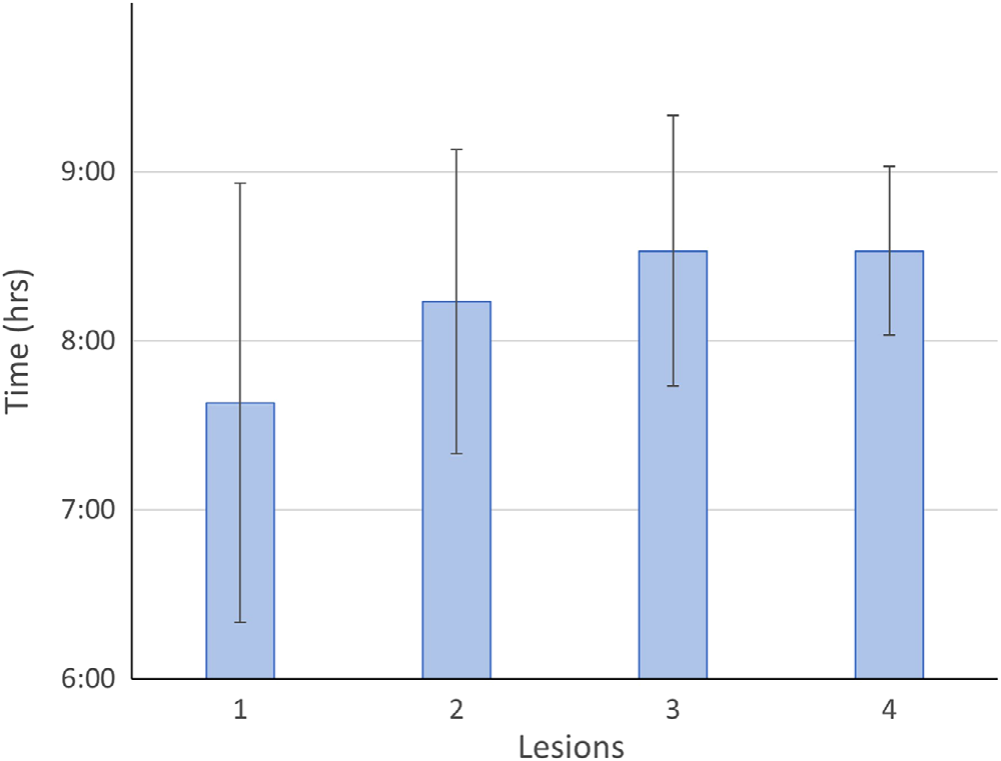
The simulation of treatment completion times as a function of lesions treated. The bars indicate the average and standard deviation.

Looking at specific factors that might affect the simulation to treatment delivery completion time, only the number of intracranial lesions trended towards significance, as shown in Figure 4. For single‐lesion cases, the entire process took on average approximately 7.5 h, while for 3–4 lesion cases it took approximately 8.5 h.

**FIGURE 5:**
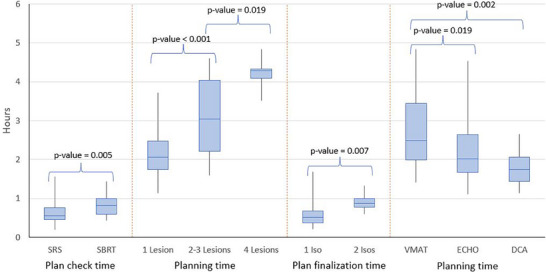
Comparisons of different times to complete certain tasks (plan check, planning, plan finalization) between different factors (treatment technique, number of lesions or isocenters, optimization) that had a significant impact on those completion times. A box plot shows the median by the horizontal line, the 1st and 3rd IQR with box bounds, and the full range with the whiskers.

We also looked at the effect of the number of lesions, type of treatment (SRS vs. SBRT), number of isocenters, and treatment technique (VMAT, DCA, ECHO, hyperarc) on the timelines. We report the average ± 1 standard deviation for the quantities presented next. We found statistically significant differences in the time it took to complete the plan check between SRS and SBRT (38 ± 17 min vs. 50 ± 17 min, *p* = 0.005). Treatment planning took on average 2.2 ± 0.6 h for 1‐lesion plans, 3.1 ± 1.0 h for 2–3 lesion plans (*p* < 0.001), and 4.2 ± 0.5 h for 4‐lesion plans (*p* = 0.02). The number of isocenters used in the SRS plans did not have a significant effect on the simulation to treatment or planning times; however, it had a significant impact on plan finalization, with single‐isocenter plans taking 35 ± 18 min to finalize, and two‐isocenter plans taking 54 ± 14 min to finalize (*p* = 0.007). Finally, in terms of the planning technique, we found that DCA SRS planning was the fastest, taking 1.8 ± 0.5 h on average, similar to ECHO IMRT SBRT auto‐planning time (2.2 ± 0.8 h, no statistical significance), while VMAT planning took 2.8 ± 0.9 h, which was significantly longer than both ECHO (*p* = 0.02) and DCA (*p* = 0.002). Plan finalization took on average 34 ± 17 min with VMAT, while it took almost 50 ± 25 min with DCA (*p* = 0.007). Figure 5 shows a visual breakdown of the time it took to complete these tasks.

Finally, to assess the quality and safety of the workflow, we examined the risk events associated with the same‐day workflow. We saw a total of 13 events related to the same‐day patients. The majority of the events were either related to a process not followed, related to scheduling and completing steps late, or to communication failures, such as documentation being incorrect or missing.

## 4 DISCUSSION

In this work, we presented our same‐day program, under which we have successfully treated SRS and SBRT patients on the same day as their simulation. We found that the majority of patients added to the program were patients with brain metastasis requiring SRS. The program was especially helpful for patients on clinical trials and protocols that needed to be treated on a specific day. The number of lesions to be treated influenced the time it took to complete the workflow. With a planned nursing visit at 7:30 AM and simulation at 8 AM, we were able to complete all the steps and finish treatment on the same day for all patients (7 PM for 95% of patients), and with no major incidents.

As Figure 3 shows, the CT simulation was on average 20 min delayed; however, those delays did not significantly correlate with later end times. Volume completion, number of volumes, and treatment planning technique were most significantly correlated with treatment completion times. The time allocated for planning was adequate for 90% of the cases; however, the time allocated for volume completion was about 1 h 30 min less than the time that was needed for 90% of the cases. Typical delays in volume completion were due to the treating radiation oncologist requiring input from a neurosurgeon. The main reason for longer planning times was treating a higher number of lesions, especially when multiple isocenters were needed. There was a clear pattern showing increasing planning times as more brain lesions were included in the SRS plan. While the goal was to limit the inclusion of patients to those with fewer than four lesions, there were cases where either more lesions were discovered when the MRI was taken, or more lesions were present, but the original intent was to only treat the most symptomatic to keep the number to fewer than four for the same‐day pathway. In certain cases, however, during the planning process, the radiation oncologist and planner decided to include four lesions in the plan either because that did not increase plan complexity, or because it would make subsequent plans to include more lesions more complicated. The majority of SRS planning was done with VMAT optimization, but DCA planning took significantly less time to complete. Therefore, for single‐lesion cases or for two lesions treated with separate isocenters, whenever possible without compromising plan quality, DCA could be used to speed up the planning process. Finally, the plan check time for SRS was significantly shorter than for extracranial SBRT. We believe that this can be attributed to many extracranial SBRT cases having prior treatment, therefore requiring the additional step of prior radiation treatment review. Handling prior treatment can be unpredictable for SBRT, depending on the body site and OARs involved. On the other hand, relevant prior treatments for intracranial SRS are usually other brain lesions that do not contribute a significant dose to each other or OARs. For all cases, the mandatory conference between the physician and planner was usually the place where re‐irradiation concerns were discussed, and feedback was given to the planner on how to proceed with planning. For complex cases with multiple prior treatments, the physician had to either allow more time for planning, disqualifying the patient for same‐day treatment workflow, or, if the patient's clinical presentation indicated significant risk with delaying treatment, a full prior treatment review with the completion of a physics consult note was waived. In those cases, it was required that the physician provide guidance on constraints that ensured the safety of radiation treatment based on a quick review of the prior records.

Many patients were excluded from the analysis since they did not follow the same‐day schedule. The main reason was that the simulation was completed on the day before the treatment day. Even though there is technically no issue with having the simulation scan completed earlier, for the purposes of this analysis, the completion times for each task would not be as expected, and our goal was to evaluate the feasibility of a same‐day workflow. In general, flexibility in the simulation time was desired, and it led to an eventual change in the timeline to allow for simulations to be done the day before, with the understanding that the rest of the workflow would not be done until the designated same‐day slot, when resources had been allocated for that purpose. We quickly realized that identifying the patients ahead of time was very important to allocating resources appropriately and keeping timelines. We therefore instituted the 1 PM cut‐off time for filling the spot the day prior to the same‐day procedure. A quick review of the MRI the day prior and a touch base with the physician via an initial conference can identify potential issues and save time later in the process. To expedite the process, often the plan checkers began reviewing volumes and fusions as soon as they were completed, both to save time later but also to identify issues before the plan was completed. Effective communication and coordination with the main stakeholders were the main issues we faced, particularly in the beginning. As a result, we established a uniform method for identifying patients for the same‐day program by using a shared calendar where anyone from the clinical teams could add a patient on a day that same‐day planning was available. It is also essential to have a main point of contact for each clinical group that is responsible for the patient's care that day. This person should be available to respond within a reasonable timeframe to minimize delays. As a teaching institution, we also encountered delays with training. It is often our practice that the medical residents work on the contours first before passing on to the attending physician for review and approval. For effective communication and hand‐offs, we created a chat with the main stakeholders of the day to keep everyone in the loop. Another important lesson learned was to ensure that the designated treatment machine for the same‐day treatments does not have any limitations that would prevent a patient from being treated on that machine. For our program, the designated machine was a high‐definition MLC machine. There were occasions where the PTV exceeded the physical limitations of the jaw size, or the treatment required deep inspiration breath hold (DIBH), in which case another machine was necessary for treatment. We now ensure that we have a backup machine both for cases with special requirements, and also if the primary machine is down. Finally, a source of delay was also identified in cases where an image fusion was not needed. Our original CarePath included a fusion task by default, which caused additional questions. To further streamline the workflow, we created a separate CarePath without the image fusion task.

Our work is not the first to explore faster simulation for treatment workflows. In fact, same‐day sim‐to‐treat workflows are becoming increasingly popular, in particular as they relate to machines capable of adaptation (MR or CBCT‐based). Nelissen et al. developed a workflow to deliver rapid palliative treatment based on pre‐planning done on a diagnostic CT.^28^ The day of treatment, a new plan is generated based on a synthetic CT generated from a CBCT. The treated 47 patients with this workflow, which was generally well‐liked by patients, and resulted in plans with improved dosimetric coverage. Schiff et al. accrued 16 patients on a prospective clinical trial to investigate an MRI‐only workflow to deliver urgent palliative radiotherapy.^29^ They showed the feasibility of the workflow without any excess toxicity. Finally, Palacios et al. developed a same‐day workflow from consultation to treatment using MR‐guided RT.^30^ They treated 10 patients with small‐cell lung cancer. All patients completed a reported experience questionnaire after treatment and resulting in high patient satisfaction. They used semi‐automated contouring and performed a pre‐planning on a diagnostic scan to speed up the process. With this work, we demonstrate how same‐day workflows for complex treatments can be feasible on conventional linacs as well.

Automation was critical in this work to be able to design a same‐day workflow, both inclusive of SRS and SBRT. While in our network we rely on in‐house scripts to automate processes, many companies are developing software solutions that aim at automating autosegmentation and planning, which are two of the most time‐consuming steps in the radiotherapy process. At our institution, our standard workflow includes OAR autosegmentation^17^ and we are in the process of adding autosegmentation for brain metastases.^31^ These AI methods help physicians to complete the often‐laborious task of delineating relevant targets and OARs. In addition, most of the timeline is spent on planning. Automated planning is becoming more popular^32^ and future developments for more complex treatments may help expedite more patients who need more complex plans. This was already demonstrated to an extent in this work, where the use of our in‐house ECHO optimization algorithm sped up planning significantly and allowed us to include extracranial metastatic lesions.^22^ Despite these developments, even more automation is needed to be able to both increase the number of cases that we can finish by our 6 PM goal and expand the use of this workflow to more complex cases. A key step that can take a long time and often contributes to delays is the simulation scan. In recent years, there have been several efforts to develop simulation‐free workflows. Eliminating the simulation step would greatly reduce the amount of time patients need to spend at the hospital on the day of treatment.^10^, ^33^, ^34^

## 5 CONCLUSION

A same‐day workflow implementation at our institution resulted in high‐quality SRS and SBRT plans for patients with intracranial or extracranial metastases. In this study, patient and treatment information were collected, as well as time information from key parts of the process. This allowed us to identify and demonstrate reasons for treatment delays. Important factors to consider when implementing such expedited workflows are careful selection of the planning technique to limit plan complexity, for example, limiting the number of lesions, using 1 isocenter and DCA, and employing automation when available, especially to complete the time‐consuming tasks of target delineation and planning. In fact, increasing automation in all parts of the process is critical, and will determine how far we can push the boundaries on the complexity of the plans that can be prepared and delivered in an expedited fashion.

## AUTHOR CONTRIBUTIONS

*Program design*: Michalis Aristophanous, Jean Moran, Sean McBride, Daniel Gomez, and Laura Cervino. *Writing manuscript*: Michalis Aristophanous, Dylan Hsu, and Laura Cervino. *Data analysis*: Dylan Hsu, Sernger Shen, Michalis Aristophanous and Dongxu Wang. *Clinical implementation oversight*: Michalis Aristophanous, Sernger Shen, Jean Moran, Ase Ballangrud, Sean McBride, Daniel Gomez, Luke Pike, Kathryn Beal, and Jonathan Yang. *Dashboard design*: Anyi Li.

## CONFLICT OF INTEREST STATEMENT

Daniel Gomez has had research grants from AstraZeneca, Johnson & Johnson, Varian, Amgen, and Summit; has received consulting fees from AstraZeneca, Regeneron, Johnson & Johnson, Olympus, Medtronic, and GRAIL; has received honoraria from Varian, Clinical GME Group, and MedLearning Group; and is in the steering committee for a trial with Johnson & Johnson. Kathryn Beal is a member of the Board of Directors for Conquer Cancer, ASCO Foundation Board, and Magnolia Medical Technologies. Laura Cervino is on the Advisory Board of Prexient Inc. Jean Moran has received grant funding from Varian; she has received Honoraria from the Connecticut area Medical Physics Society; has a patent on combined radiation acoustics and ultrasound for radiotherapy guidance and cancer targeting; is co‐founder and board member of Prexient Inc, she is the chair of the work group on the Science Council EDI, and Report Writing of the American Association of Physicists in Medicine; she is the Vice Chair of the Research Committee of the Radiation Oncology Institute, and the Co‐Chair of the Radiation Oncology Safety Stakeholders Initiative; she is a consultant to the Michigan Radiation Oncology Quality Consortium, and had licensed software to FuseOncology. Dongxu Wang has received a licensing fee or royalties from Ion Beam Applications S.A.; and a consulting fee from Mevion Medical Systems, Inc. Luke Pike has received funding from Delfi Diagnostics, Varian Life Sciences, Caris Life Sciences, Harbinger Health, Genece Health, and the Department of Defense; and he has consulting agreements with Monograph Capital, Genece Health, and DxCover Rx.
